# History of the discovery of the malaria parasites and their vectors

**DOI:** 10.1186/1756-3305-3-5

**Published:** 2010-02-01

**Authors:** Francis EG Cox

**Affiliations:** 1 Department of Infectious and Tropical Diseases, London School of Hygiene and Tropical Medicine, London WC1E 7HT UK

## Abstract

Malaria is caused by infection with protozoan parasites belonging to the genus *Plasmodium *transmitted by female *Anopheles *species mosquitoes. Our understanding of the malaria parasites begins in 1880 with the discovery of the parasites in the blood of malaria patients by Alphonse Laveran. The sexual stages in the blood were discovered by William MacCallum in birds infected with a related haematozoan, *Haemoproteus columbae*, in 1897 and the whole of the transmission cycle in culicine mosquitoes and birds infected with *Plasmodium relictum *was elucidated by Ronald Ross in 1897. In 1898 the Italian malariologists, Giovanni Battista Grassi, Amico Bignami, Giuseppe Bastianelli, Angelo Celli, Camillo Golgi and Ettore Marchiafava demonstrated conclusively that human malaria was also transmitted by mosquitoes, in this case anophelines. The discovery that malaria parasites developed in the liver before entering the blood stream was made by Henry Shortt and Cyril Garnham in 1948 and the final stage in the life cycle, the presence of dormant stages in the liver, was conclusively demonstrated in 1982 by Wojciech Krotoski. This article traces the main events and stresses the importance of comparative studies in that, apart from the initial discovery of parasites in the blood, every subsequent discovery has been based on studies on non-human malaria parasites and related organisms.

## Background

Malaria is an ancient disease and references to what was almost certainly malaria occur in a Chinese document from about 2700 BC, clay tablets from Mesopotamia from 2000 BC, Egyptian papyri from 1570 BC and Hindu texts as far back as the sixth century BC. Such historical records must be regarded with caution but moving into later centuries we are beginning to step onto firmer ground. The early Greeks, including Homer in about 850 BC, Empedocles of Agrigentum in about 550 BC and Hippocrates in about 400 BC, were well aware of the characteristic poor health, malarial fevers and enlarged spleens seen in people living in marshy places. For over 2500 years the idea that malaria fevers were caused by miasmas rising from swamps persisted and it is widely held that the word malaria comes from the Italian mal'aria meaning spoiled air although this has been disputed. With the discovery of bacteria by Antoni van Leeuwenhoek in 1676, and the incrimination of microorganisms as causes of infectious diseases and the development of the germ theory of infection by Louis Pasteur and Robert Koch in 1878-1879, the search for the cause of malaria intensified. Scientific studies only became possible after the discovery of the parasites themselves by Charles Louis Alphonse Laveran in 1880 and the incrimination of mosquitoes as the vectors, first for avian malaria by Ronald Ross in 1897 and then for human malaria by the Italian scientists Giovanni Battista Grassi, Amico Bignami, Giuseppe Bastianelli, Angelo Celli, Camillo Golgi and Ettore Marchiafava between 1898 and 1900. Excellent histories of this disease include those by Celli [1], Stephens [2], Scott [3], Russell [4], Foster [5], Garnham [6,7], Harrison [8], Bruce-Chwatt [9], Desowitz [10], McGregor [11], Poser & Bruyn [12] and Schlagenhauf [13].

## The life cycle of the malaria parasites, *Plasmodium *spp

**Figure 1:**
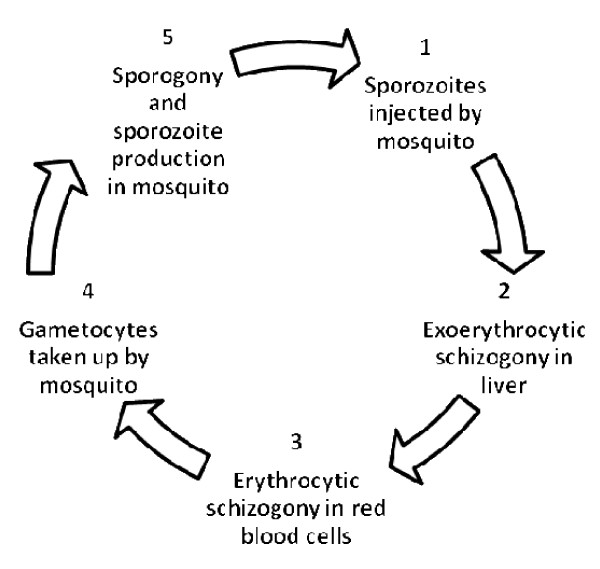
**Schematic life cycle of *Plasmodium *spp**.

In order to understand the historical events it is necessary to summarise briefly our current state of knowledge. Malaria is caused by infection with five species of *Plasmodium *the life cycles of which are very similar (Figure 1).

Infection begins when (1) sporozoites, the infective stages, are injected by a mosquito and are carried around the body until they invade liver hepatocytes where (2) they undergo a phase of asexual multiplication (exoerythrocytic schizogony) resulting in the production of many uninucleate merozoites. These merozoites flood out into the blood and invade red blood cells where (3) they initiate a second phase of asexual multiplication (erythrocytic schizogony) resulting in the production of about 8-16 merozoites which invade new red blood cells. This process is repeated almost indefinitely and is responsible for the disease, malaria. As the infection progresses, some young merozoites develop into male and female gametocytes that circulate in the peripheral blood until they are (4) taken up by a female anopheline mosquito when it feeds. Within the mosquito (5) the gametocytes mature into male and female gametes, fertilization occurs and a motile zygote (ookinete) is formed within the lumen of the mosquito gut, the beginning of a process known as sporogony. The ookinete penetrates the gut wall and becomes a conspicuous oocyst within which another phase of multiplication occurs resulting in the formation of sporozoites that migrate to the salivary glands of a mosquito and are injected when the mosquito feeds on a new host.

## The malaria parasites

Our understanding of the life cycle of the malaria parasites did not proceed in the logical order just outlined but more like a jigsaw in which the various pieces were painstakingly put into place and, like a jigsaw, often involved mistakes and false starts. The story begins with the discovery of the stages in the blood. Many textbooks merely state that 'in 1880 Laveran discovered the malaria parasite' words that do not give this discovery the credit it deserves. In order to understand the background of this discovery it is necessary to go back to the 1870s. The discoveries of Pasteur and Koch had precipitated a search for a bacterial cause for many diseases including malaria. By 1879 the miasma theory was going out of favour and the two theories vying for contention were whether the microorganisms responsible were transmitted (1) by air and inhalation or (2) by water and ingestion. The leading theory was that proposed by the Italian Corrado Tommasi-Crudeli and the German, Theodor Albrecht Edwin Klebs, an eminent microbiologist who had been the first person to see the bacteria responsible for typhoid and diphtheria. Tommasi-Crudeli and Klebs claimed that they had isolated from the waters of the Pontine Marshes, where malaria was prevalent, a bacterium, *Bacillus malariae*, which when isolated in culture and injected into rabbits caused febrile infections accompanied by enlarged spleens reminiscent of malaria [14]. It was against this background that Charles Louis Alphonse Laveran, an unknown French army officer working in Algeria, challenged the perceived wisdom and began in his own words 'to follow the pigment'. Beginning with the known fact that the spleens of malaria patients contained pigment he began to look for pigment in the fresh unstained blood of patients and observed it first in leucocytes and then in or on red blood cells. Looking more carefully, he observed several different forms of erythrocytic organism including crescents, spherical motionless bodies with pigment, spherical moving bodies with pigment and bodies that extruded flagella-like structures all of which he thought were on the outside of the red cells. These observations are particularly interesting because Laveran not only used fresh blood but also a dry objective with a maximum magnification of ×400 diameters. He also suggested a course of events that began with clear spots that grew, acquired pigment and filled the corpuscle which then burst coinciding with the fevers associated with malaria. Laveran meticulously examined the blood of 200 patients and in 148 observed the crescentic bodies in all cases of malaria but never in those without malaria. He also noted that quinine removed these stages from the blood. Laveran quickly realised that he had found a parasitic protozoan which he called *Oscillaria malariae*. He presented his findings to the French Academy of Medical Sciences in December 1880 [15] but failed to persuade any of the eminent microbiologists, zoologists or malariologists of the day that he was seeing anything other than disintegrating red blood cells. Nevertheless he persevered and by 1884 had convinced the leading Italian malariologists including Bignami, Golgi and Marchiafava that malaria was caused by a protozoan and not a bacterium [16]. His biggest triumph came in the same year when he also convinced the more cynical microbiologists Louis Pasteur, Charles Edouard Chamberland and Pierre Paul Émile Roux. Robert Koch, one of the most influential microbiologists of his time, however, remained sceptical until 1887. Nevertheless in some quarters the miasma theory persisted and as late as 1895 the American R. C. Newton, a supporter of Tommasi-Crudeli, wrote that 'Aerial and aquatic transportation of malaria has been proved' [17]. (This paper is worth reading in full because, although based on what we now know to be false premises, it contains a mass of interesting information about the prevention of malaria such as the use of screens or mosquito nets to exclude insects, closing doors at night and lighting fires out of doors). Laveran was awarded the Nobel Prize for Medicine in 1907 and his discoveries are described in some detail by the Sergent brothers [18] and Bruce-Chwatt [19] as well as in the various histories of malaria listed above.

What was remarkable about Laveran's discovery was that it was without precedent as no protozoan had previously been found inhabiting any kind of human blood cell. Unbeknown to Laveran or the Italian malariologists, however, the Russian physiologist, Vassily Danilewsky had been examining the blood of birds and reptiles in the Ukraine and had discovered a number of parasites including trypanosomes and others that he identified as 'pseudovacules'. Anyone who has studied blood parasites will immediately recognise his description of 'pseudovacuoles' as unstained malaria parasites. By 1885 Danilewsky had recognised the three most common genera of intraerythrocytic blood parasites of birds now known as *Plasmodium*, *Haemoproteus *and *Leucocytozoon *but, as he had published much of his work in Russian, it was not until his three volume book *La Parasitologie Comparée du Sang *had been published in French in 1889 that this information became widely available [20]. Thereafter there began searches for other malaria parasites in reptiles, birds and mammals and this was facilitated by the accidental discovery of a methylene blue-eosin stain by Dimitri Leonidovitch Romanowsky in 1891 [6]. Romanowsky's stains became popular at the beginning of the twentieth century and remain the basis of blood stains such as Leishman's, Giemsa's and Wright's to the present day. These stains colour the nucleus of the parasite red and the cytoplasm blue permitting their easy identification and are used not only for malaria parasites but also for trypanosomes, leishmanias and filarial worms. Romanowsky's discovery is one of the most significant technical advances in the history of parasitology.

Meanwhile the Italian workers, now convinced that malaria was caused by a parasite, took up the challenge with vigour and Marchiafava and Bignami, using a combination of eosin-based blood stains and the oil-immersion microscope objective developed by the Carl Zeiss Company in 1882-4, observed amoeboid movement of the organism. This left them in no doubt that they were dealing with a protozoan parasite that invaded red blood cells, grew within the cells and produced daughter cells that invaded fresh blood cells [21]. Thereafter the Italian views dominated malaria research and, based on observations of the erythrocytic stages of the parasite, Golgi between 1885-6 differentiated between tertian (48 hour periodicity) and quartan (72 hour periodicity) malaria [22] and in 1889-1890 Golgi and Marchiafava further described the differences between mild Spring malaria (benign tertian) and severe Summer-Autumn (malignant tertian) malaria [23].

By this time it had also become clear that the paroxsms characteristic of malaria coincided with the bursting of infected red blood cells and the release of the products of multiplication something that Laveran, who had also realised that in the case of malignant tertian malaria the brain was involved, had proposed [24]. Thus by 1890 it was known that malaria was caused by a protozoan parasite that invaded and multiplied in red blood cells and, after a lot of confusion, that there were three species with specific periodicities and other characteristics responsible for benign tertian (*Haemamoeba vivax*), malignant tertian (*Laverania malariae*) and quartan (*Haemamoeba malariae*) malaria now respectively *Plasmodium vivax*, *P. falciparum *and *P. malariae*. The situation as it existed in 1900 is beautifully summarised by Grassi in his monograph, *Studi di uno Zoologo Sulla Malaria *[25] and, although more details have since been added, this work remains as relevant today as it was 110 years ago. In 1918, John Stephens, working in West Africa, discovered a fourth species which resembled *P. vivax *which he described as *P. ovale *in 1922 [26].

## The sexual stages

The action now moves to Canada in 1897 and to the United States a year later where a medical student, William MacCallum, and his colleague, Eugene Opie, while examining the blood of crows infected with *Haemoproteus columbae*, a haematozoan closely related to the malaria parasites, observed flagellated structures which they described in detail and also recorded how the flagellated bodies fused with non-motile bodies to form a vermicule (now called an ookinete) [27]. MacCallum suggested that he was witnessing sexual reproduction that paralleled that in mammals (and, it should be noted, related sporozoans that were already familiar to European zoologists [28,29]) and that the flagellated forms were male gametes, the non-motile forms female gametes and the vermicule the zygote. MacCallum's findings are very significant as he realised that: 'Have we not here...a sexual process...the result of which is the motile vermiculus? This is a process which we might have expected and which I am confident will be found to occur in the case of the human malaria parasites...' [30]. The significance of this observation initially eluded Ronald Ross (see below) something that remained with him for the rest of his life [31] but was not missed by Patrick Manson who wrote to Ross that 'MacCallum's observation on *Halteridium*; if it is correct, it is of the greatest importance' [31,32]. Here MacCallum reached a dead end because he believed that the vermicule then invaded cells of the vertebrate host but was unable to pursue this line of investigation.

## Transmission

Despite all their accumulated knowledge and skills no malariologists could explain how the parasite spread from one human to another. The clues were, however, in place. Over the centuries, circumstantial evidence had accumulated that suggested that mosquitoes might somehow be connected with malaria and by 1883 the American physician, Albert King, had assembled the mass of evidence that was to become known as the mosquito-malaria doctrine [33]. Between 1884 and 1897, Laveran, Manson (who in 1877 had demonstrated that the filarial worms responsible for lymphatic filariasis were transmitted by mosquitoes [34]), and the Italian malariologists, had become increasingly convinced that mosquitoes were involved in the transmission of malaria. Thereafter opinions differed with some observers, including Manson, believing that humans became infected by drinking water contaminated by infected mosquitoes while others thought that the infection was acquired by inhaling dust from dried-up ponds in which infected mosquitoes had died, in other words, variations on the water and ingestion and air and inhalation theories proposed by Tommasi-Crudeli and Klebs in 1879. Manson also toyed with the idea that transmission might be mechanical, i.e. the parasites were passively carried from host to host on the proboscis of a mosquito.

**Figure 2:**
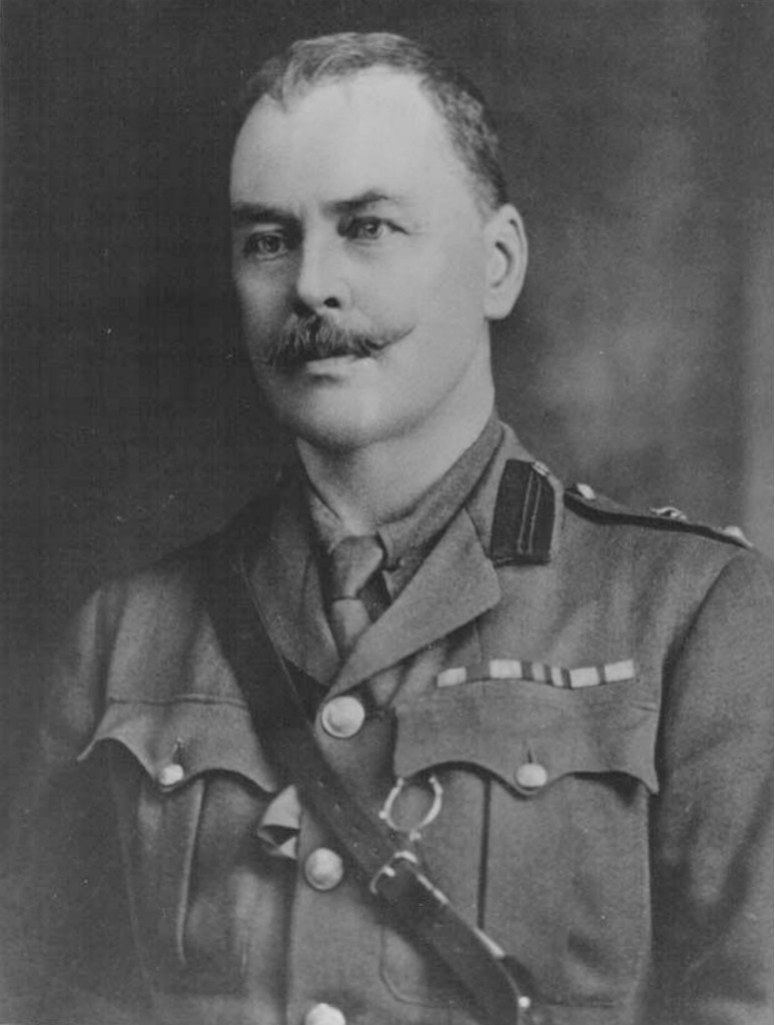
**Ronald Ross 1857-1932**. Photograph by courtesy of the Royal Society of Tropical Medicine and Hygiene. Portraits of other scientists who were involved in the elucidation of the life cycle of the malaria parasites can be found elsewhere [9,40].

By 1894 Manson, who had spent much of his working life in Taiwan and was then in his 50s and in an established medical practice in London, turned his attention to the possibility of mosquito transmission of malaria but, as he was unable to go to malarious countries himself, he needed someone to carry out the necessary investigations and experiments for him. His colleague-to-be was an unlikely choice, Ronald Ross (Figure 2).

Ronald Ross 1857-1932. In 1897 Ronald Ross working in India discovered that culicine mosquitoes transmitted the avian malaria parasite *Plasmodium relictum *and suggested that human malaria parasites might also be transmitted by mosquitoes. Later, when working in Sierra Leone in 1899, he demonstrated that the human malaria parasites were indeed transmitted by anopheline mosquitoes. In the meantime, however, several Italian scientists had already shown that this was the case.

Ross then aged 37 was an established army surgeon working in India who did not believe that malaria was caused by a blood parasite but thought that it was an intestinal infection. Throughout the second half of 1894, Manson worked on Ross, showed him blood slides containing malaria parasites and convinced him that incriminating a mosquito vector of malaria was a goal worth aiming for. Ross returned to India and over the next four years Manson directed operations at a distance and we are fortunate to have an almost complete collection of the letters that passed between the two men [32]. This was not an easy relationship partly because Ross's first priorities were his military commitments and these inevitably delayed the work he was doing with malaria and partly because, from time to time, Ross seemed more interested in writing poetry and novels. Nevertheless, the cooperation reached a satisfactory conclusion but later ended in acrimony.

Manson, who had access to malaria patients in London, had observed that it was only when blood taken from such patients began to cool that the flagellated forms and subsequent fertilization, as described by MacCallum, appeared and concluded that further development must occur outside the human body in another host, probably a mosquito. Ross, having returned to India, examined several thousand mosquitoes from endemic areas without any success but, remembering Laveran's dictum 'follow the pigment' and Manson's advice to 'follow the flagellum', a reference to the flagella of the male gamete, he eventually found pigmented bodies, which he called spores, on the stomach wall of a mosquito experimentally fed on an infected patient. Ross was no entomologist (in fact the only book he had on entomology was one written for anglers) so he classified the mosquitoes he was studying as grey or barred-back (A), brindled (B), and dappled-winged (C). We now know that the grey mosquitoes were culicines and that the dappled-winged mosquitoes were anophelines. Grey mosquitoes were very common but never contained the pigmented spores. On the other hand the rarer 'dapple-winged' mosquitoes, after being fed on a malaria patient, were found to contain pigmented bodies that ruptured releasing 'rods' that invaded the mosquito's salivary glands. Ross had now made the crucial break-through and had found developmental stages of human malaria parasites in anopheline mosquitoes and, in his letters, he calls August 20th 1897 'Mosquito day' [31,32]. Ross was on the brink of demonstrating that anopheline mosquitoes could transmit human malaria but unfortunately he was not able to complete his studies because at this crucial stage he was posted to Calcutta where there was very little malaria [31]. He did, however, have access to laboratory facilities and, remembering that in 1894 Manson had mentioned the possibility of using malaria parasites of birds in his investigations, he turned his attention to an avian malaria parasite, *Proteosoma relictum *(now called *Plasmodium relictum*), common in many species of birds including crows and sparrows. This parasite, he discovered, was transmitted by his 'grey' (culicine) mosquitoes, probably *Culex fatigans*. Of 242 'grey' mosquitoes fed on infected birds, 178 developed pigmented spores. Ross concluded that mosquitoes fed on infected birds took up male and female gametocytes which fertilized in the mosquito gut and developed into spores on the surface of the mosquito's gut within which rod-like structures were produced that invaded the mosquito's salivary glands and were injected into a new host when the infected mosquito fed. His results were made public in 1898 [35,36]. Ross surmised correctly that human malaria was probably transmitted in the same way and later wrote that 'The triumph of 20 August was now completed and crowned by that of 9 July 1898' [31]. These experiments finally convinced Manson, that malaria was transmitted through the bite of a mosquito contrary to his earlier opinion that the infective stages were discharged into water. He nevertheless still thought that discharge of infective stages into water was the way that filiarial worms were transmitted until it was shown that they too were transmitted via the bite of a mosquito by George Carmichael Low in 1900 [37].

Although Ross had elucidated the whole of life cycle of *Plasmodium relictum *in culicine mosquitoes and had come tantalizingly close to completing the mosquito stages of the human malaria parasites the actual proof of the transmission of human malaria by anopheline mosquitoes still remained unresolved. Ross recorded that one single experiment could bring about the life cycle of human malaria [31] but his military duties took precedence and he was sent to work on an epidemic of plague that was then spreading across India and was not allowed to test his hypothesis because of the plague.

In the meantime several Italian workers were already on the trail. Bignami had suggested in 1896 that mosquitoes might transmit malaria by inoculation but it wasn't until 1898 that he and Grassi, who were fortunate to have access to sites where malaria was present near Rome and in Sicily, produced the final proof when they fed local *Anopheles claviger *mosquitoes on infected patients and subsequently transmitted the infection to uninfected individuals via the bite of these mosquitoes [38]. Over the next two years the Italians proved that only female *Anopheles *mosquitoes could transmit malaria and methodically consolidated their findings and described the whole blood-mosquito life cycles of *P. vivax*, *P. falciparum *and *P. malariae *(see Grassi's classical monograph, *Studi di uno Zoologo Sulla Malaria *[25]). Ross, in the meantime, had been posted to Sierra Leone where within a few weeks after his arrival in 1899 he had demonstrated the development of *P. falciparum, P. vivax *and *P. malariae *in anopheline mosquitoes. Meanwhile, in London, Manson persuaded Bignami and Bastianelli to send him *A. maculipennis *mosquitoes infected with benign tertian malaria which he used to infect his medial student son, Patrick Thurburn, and another volunteer thus completing this part of the story. More detailed accounts of these discoveries can be found in reviews by Ascenzi [39], Dobson [40] and Fantini [41] which were published together in the proceedings of a meeting held in Rome to commemorate one hundred years of malariology.

The discovery of the role of mosquitoes in the transmission of malaria provided malariologists with a new weapon against this ancient disease. In a classical experiment, Grassi dispatched 112 volunteers to the Capaccio Plains, a malarious area in Italy, protected them from mosquito bites between dusk and dawn and found that only five succumbed to the disease compared with 415 unprotected volunteers who all contracted malaria [25]. Thus the possibility of controlling the disease by reducing contact with infected mosquitoes had been demonstrated. Over the next decades, methods to prevent mosquito biting by avoidance, screening and mosquito proofing dwellings and anti mosquito measures such as by the use of oils and larvivorous fish and draining mosquito habitats had become commonplace [9].

One surprising aspect of this whole story is that some of the clues about arthropod-transmission of blood-inhabiting protozoa were available several years before Ross and the Italian scientists began their investigations. In 1890 the American microbiologists Theobald Smith and Frederick Kilborne had observed that young ticks taken from cattle infected with the piroplasm *Babesia bigemina*, an intraerythrocytic protozoan resembling a malaria parasite, could infect susceptible animals and this was confirmed in a series of meticulously controlled experiments over the next two years [42]. It is strange that none of the participants in the malaria story seemed to be aware of these discoveries, probably because they were published in an American Government Agricultural document. How differently things might have turned out if they had been aware of these discoveries is a matter of speculation.

## Exoerythrocytic development

The life cycle in humans, however, remained incompletely understood and nobody knew where the parasites developed during the first 10 days or so after infection during which they could not be seen in the blood. Grassi was the first to suggest that there must be some developmental stage in cells other than red blood cells, possibly white blood cells [25]. This theory was elaborated by Grassi and his colleagues from1893 and 1894 onwards but was later abandoned mainly due to too much reliance on a mistake by the influential German scientist Fritz Schaudinn who, in 1903, described the direct penetration of red blood cells by the infective sporozoites of *P. vivax *[43]. No one else was able to confirm these observations and the phenomenon is now referred to among malariologists unkindly as 'Schaudinn's fallacy'. Nevertheless Schaudinn's ideas were adopted by such authorities as Grassi and dominated scientific opinion for over forty years. Meanwhile evidence that there was a phase of multiplication preceding that in the blood was accumulating from another source, the avian malarias. MacCallum in 1898 had observed developmental stages of *P. relictum *in the liver and spleen of infected birds [30] and thereafter there were numerous somewhat inadequate descriptions of exoerythrocytic development of a number of avian malaria parasites [6,44]. In 1937 Sydney James and Parr Tate conclusively demonstrated that in sporozoite-induced *P. gallinaceum *infections in chickens there was phase of multiplication between the injection of sporozoites and the appearance of parasites in the blood and that this occurred in cells of the reticuloendothelial system [45].

By the late 1930s there was no doubt that in all the avian malaria parasites studied there was a phase of multiplication in various nucleated cells before (and after) parasites appeared in the blood and over the next decade the complete life cycles of a number of avian *Plasmodium *and *Haemoproteus *species, differing only in detail particularly relating to the types of cells involved which varied from species to species, had been worked out. What happened in primates was not so clear and during the 1930s and 1940s there were sporadic reports of parasites in the tissues, particularly in the brain and nervous system, of animals infected with primate and bat malarias. After the end of the Second World War in 1945 malaria research throughout the world intensified and a number of workers became convinced that that there must be an exoerythrocytic stage in the life cycle of the primate malarias but what form this took was not known. This question was not resolved until 1947 when Henry Shortt and Cyril Garnham, working in London, showed that a phase of division in the liver preceded the development of parasites in the blood [46]. The crucial clues came from studies on *Hepatocystis kochi*, another parasite of monkeys first identified by Laveran as *Haemamoeba kochi*. *Hepatocystis *spp. are related to malaria parasites but do not have an erythrocytic stage in their life cycles so these parasites must have only an exoerythrocytic stage which in *H. kochi *is in the parenchyma cells of the liver [47]. This suggested to Shortt, Garnham and their colleagues that the liver might be the place to look for the elusive exoerythrocytic stages of primate malaria parasites and selected *P. cynomolgi *in rhesus monkeys for their investigations. Previous attempts by other workers had failed to find any liver forms so Shortt and Garnham decided to use 500 infected *A. maculipennis atroparvus*, a massive dose of sporozoites, and found exoerythrocytic stages seven days later [48]. Shortly afterwards Shortt, Garnham and their co-workers found exoerythrocytic forms of *P. vivax *in human volunteers [49] and subsequently in volunteers infected with *P. falciparum *in 1949 [50] and *P. ovale *in 1954 [51]. In the meantime the same team had also demonstrated exoerythrocytic stages of *P. inui*, a quartan form of primate malaria. The exoerythrocytic stages of *P. malariae *were more elusive and it was not until 1960 that Robert (Bill) Bray demonstrated their presence in experimentally infected chimpanzees [52]. The story of the discovery of the exoerythrocytic forms of malaria parasites until 1957 is told in some detail by Bray [44] and updated until 1966 by Garnham [6].

The story of the life cycle of the human malaria parasites was almost complete and had taken nearly 70 years to elucidate. There remained, however, one further question; what caused the long prepatent period between infection and the appearance and reappearance of parasites in the blood seen in some temperate strains of *P. vivax*? This led to the discovery of dormant exoerythrocytic stages, hypnozoites, by Wojciech Krotoski, working with Garnham's team, in 1982 [53].

## Other malaria parasites

It has already been noted that malaria-like parasites are commonly found in birds, mammals and reptiles and studies on many of these have contributed to our overall understanding of human malaria. Malaria-like parasites belonging to the genus *Hepatocystis *in non-human primates were first recognised by Laveran in1899 but true malaria parasites, *Plasmodium *spp., were not identified with certainty until 1907 with the independent discoveries of *P. cynomolgi*, *P. inui*, and *P. pitheci *in monkeys imported into Germany from Java [6]. Throughout the 1920s and 1930s there were increasing numbers of reports of new species from wild-caught primates including *P. knowlesi *in 1932 [6,54]. During the 1960s, there were occasional reports of accidental infections with *P cynomolgi*, *P. inui *and *P. knowlesi *in humans suggesting that some primates might act as reservoirs for human malaria but it appeared that the chances of such naturally acquired infections were very remote. However it is now known that humans are at risk from infection with *P. knowlesi*, a malaria parasite with a 24 hour erythrocytic cycle, in Southeast Asia where its natural hosts are macaque and leaf monkeys. Until 1971 there had only been two authenticated cases of naturally acquired human infections with *P. knowlesi *both in peninsular Malaysia. No other cases were recorded until 2004 when a focus of human infections was identified in Sarawak, Malaysian Borneo [55]. Since then there have been several hundred reports of human infections in the region and there is now overwhelming evidence that *P. knowlesi *is a zoonosis involving macaque (*Macaca *spp.) and leaf (*Presbytis *spp.) monkeys as reservoir hosts with mosquitoes belonging to the Leucosphyrus group of *Anopheles *mosquitoes as the vectors in Malaysia and elsewhere in Southeast Asia [56]. Retrospective examination of blood films and the application of the polymerase chain reaction (PCR) and other molecular techniques have revealed that a number of malaria cases previously attributed to *P. malariae *in Malaysia were misidentified and that they were in all probability due to *P. knowlesi *[57].

The first avian malaria parasites were discovered at about the same time as the human species and there are now about 24 species including *P. relictum*, which has contributed most to our understanding of the transmission of human malaria parasites, and *P. gallinaceum *which not only contributed to our understanding of the exoerythrocytic phases of the malaria life cycle but also, because it could easily be maintained and mosquito-transmitted in chickens, served at the main model for chemotherapeutic studies until the discovery of rodent malarias.

The first rodent malaria parasite, *P. berghei*, was identified and isolated from wild rodents in Central Africa by Ignace Vincke and Marcel Lips in 1948 and subsequently adapted to mice, rats, hamsters and gerbils and easily maintained laboratory-bred mosquitoes such as *A. stephensi *[58]. Since then three other species, *P. yoelii, P. vinckei *and *P. chabaudi*, of which there are a number of subspecies and strains, have been identified, isolated and adapted to laboratory rodents and have become the mainstay of studies on chemotherapy and have served as surrogate models of human malaria in the fields of immunology, genetics, molecular biology and biochemistry [59].

## *In vitro *cultivation

One of the most important breakthroughs in malaria research was the development of techniques that enabled scientists to grow the erythrocytic stages of malaria parasites in continuous culture pioneered by William Trager and J.B. Jensen [60] thus freeing investigators from the need to use animals for chemotherapeutic and biochemical studies. The importance of this discovery cannot be overemphasised. For the first time, scientists had access to unlimited quantities of human malaria parasites, particularly *P. falciparum*, thus reducing their dependence on laboratory animals and blood taken from humans. The ease with which the erythrocytic stages could be grown in bulk made it possible not only to test the effects of drugs directly but also to isolate and purify parasite components in order to identify biochemical pathways and molecules of potential use in the development of vaccines and chemotherapy. The cultivation of sexual stages provided insights into the genetics of human malaria parasites and the development of drug resistance. The cultivation of liver stages, although more difficult to achieve, made it possible to develop and test drugs against these stages and provided vital information about the immune responses in the liver. Finally, the cultivation of sporogonic stages has enabled scientists to discover what happens to the parasite in its mosquito vector.

## The final [?] step

The final stage in the story of our understanding of the malaria parasites that began when an unknown French scientist, working by himself in Algeria with a crude microscope, noticed that the blood of patients suffering from malaria contained organisms that he identified as parasitic protozoa culminated 122 years later when a massive team of investigators determined the compete genome of *Plasmodium falciparum *[61] since when the genomes of other malaria parasites have also been published [62].

## Conclusions

Over a century later it seems appropriate to attribute the various discoveries concerning the malaria parasites and their transmission as follows. Laveran was the first person to find parasites in the blood of patients infected with malaria in 1880, MacCullum was the first to observe the sexual stages of a malaria-like parasite, *Haemoproteus columbae*, in birds in 1897, Ross was the first to show that any malaria parasite, in this case the avian *Plasmodium relictum*, was transmitted by the bite of infected mosquitoes in 1897 and, by implication, that this would be the case for human malarias and in 1898 Grassi, Bignami and Bastienelli were the first to demonstrate that human malaria parasites were actually transmitted in this way. The most far-reaching discovery made by Ross, and one that is frequently ignored, was that a blood-sucking insect could not only take up infective organisms from an infected individual but could also transmit them some time later when it fed on an uninfected host something that was completely contrary to the received opinion of the time. It took a long time before other investigators realised the universal importance of this discovery and it was not until the first decades of the twentieth century that diseases such as African trypanosomiasis, leishmaniasis, filariasis and loaiasis were discovered to be transmitted by the bites of infected insects. This discovery was not missed by virologists who, after the discovery of viruses, soon established the concept of arthropod-borne or arboviruses or by bacteriologists looking for the mode of transmission of the plague bacillus. The tissues stages in the blood were discovered half a century later, in 1947, by Shortt and Garnham and the final mystery, the persistence of liver stages was established by Krotoski in 1962. The story of the elucidation of the complex life cycle of the malaria parasites was only possible because the various scientists involved were able to transfer knowledge gleaned from non-human malarias in birds and primates to the problem of human malaria thus emphasising the importance of comparative studies in the investigation of human diseases.

## Competing interests

The author declares that they have no competing interests.
